# Xanthogranulomatous Pyelonephritis Mimicking a Complex Renal Cyst: A Report of a Rare Case

**DOI:** 10.7759/cureus.69233

**Published:** 2024-09-11

**Authors:** Shantanu Chandrashekhar, Senthil Kumar, Saravanan Jambunathan, Anurag Sahu, Balaji Radhakrishnan

**Affiliations:** 1 Urology, Sri Ramaswamy Memorial (SRM) Medical College Hospital and Research Centre, Chennai, IND; 2 Pathology and Laboratory Medicine, Sri Ramaswamy Memorial (SRM) Medical College Hospital and Research Centre, Chennai, IND

**Keywords:** complex renal cyst, focal xgpn, renal pseudotumor imaging, urology, xanthogranulomatous pyelonephritis

## Abstract

Xanthogranulomatous pyelonephritis (XGPN) is a chronic granulomatous inflammatory condition that affects the kidney and can often be hard to diagnose preoperatively due to its varying clinical presentations. We present here a rare case of a 36-year-old man with focal XGPN with preoperative CT imaging showing a large heterogenous lesion (computer tomography Hounsfield unit (CT HU) + 20) of size ~ 8.6 x 8.9 x 9.4 cm (anteroposterior (AP) x transverse (TR) x craniocaudal (CC)) arising from the inter pole region with few hyperdense solid components and septations suggestive of a complex renal cyst. The patient underwent open decortication and excision of the infected complex right renal cyst with right DJ stenting. He was treated with antibiotics and had an uneventful post-operative period.

## Introduction

Xanthogranulomatous pyelonephritis (XGPN) is a chronic granulomatous inflammatory process affecting the kidney [1]. XGPN is a mimicker of multiple diseases like complex renal cysts, renal cell carcinoma, and tuberculosis of the kidney due to its varying clinical presentations. Usually, patients present with loin pain, fever, and loss of weight and appetite. Although it is a histological diagnosis, CT imaging has signs and can guide us to the appropriate diagnosis of XGPN, like diffuse kidney involvement, dilated calyces, presence of obstruction and calculi in the renal pelvis, often “bear’s paw” signs can be noted [2]. Here, we present a case who had loin pain and fever, but imaging did not show any classical XGPN features. The diagnosis was confirmed postoperatively on histopathologic examination as XGPN.

## Case presentation

A 36-year-old male patient presented to the emergency room with chief complaints of right loin pain for three months. The pain was intermittent, mild, dull aching, and increased in intensity since one day. He initially sought treatment from a general practitioner and was started on intermittent antibiotics. He had a history of a mild fever since one day. There was no history of nausea or vomiting. There was no history of hematuria, dysuria, or burning micturition. He had no history of urolithiasis in the past. He had no known comorbidities and no history of any prior surgical intervention. He denied a history of trauma.

Physical examination revealed right renal angle tenderness. Laboratory test results of complete blood count, serum urea, creatinine, liver function, and coagulation profile were normal. ESR (erythrocyte sedimentation rate) was mildly elevated at 36mm/hr. Urine culture was positive for Enterococcus. Urine was negative for acid-fast bacillus.

**Figure 1:**
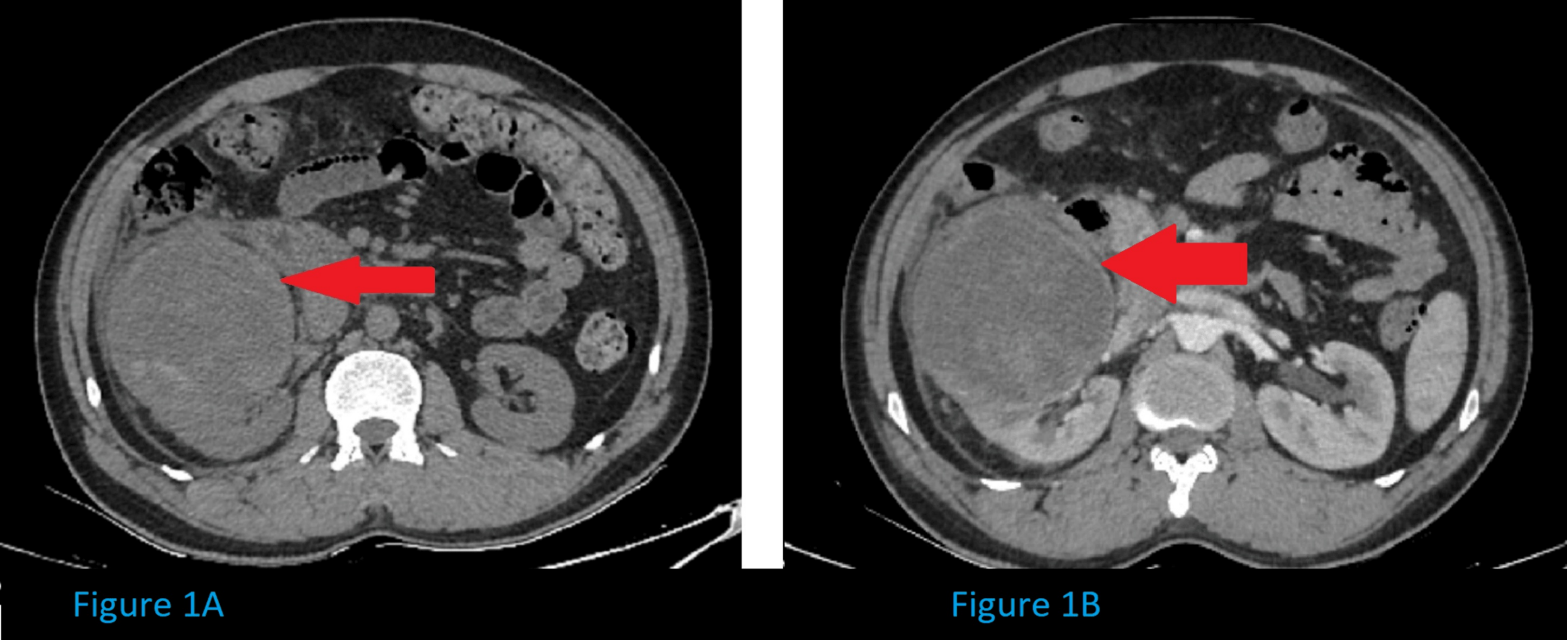
Non-contrast CT scan images (1A) Shows a large heterogenous lesion arising from the inter pole region with few hyperdense solid components and septations within. (1B) The contrast-enhanced computed tomography (CECT) image shows no significant enhancement of the lesion.

As shown in Figure 1, computer tomography (CT) revealed a large heterogenous lesion (computer tomography Hounsfield unit (CT HU) + 20) of size ~ 8.6 x 8.9 x 9.4 cm (anteroposterior (AP) x transverse (TR) x craniocaudal (CC)) arising from the inter pole region with few hyperdense solid components and septations within. No significant contrast enhancement was noted.

**Figure 2:**
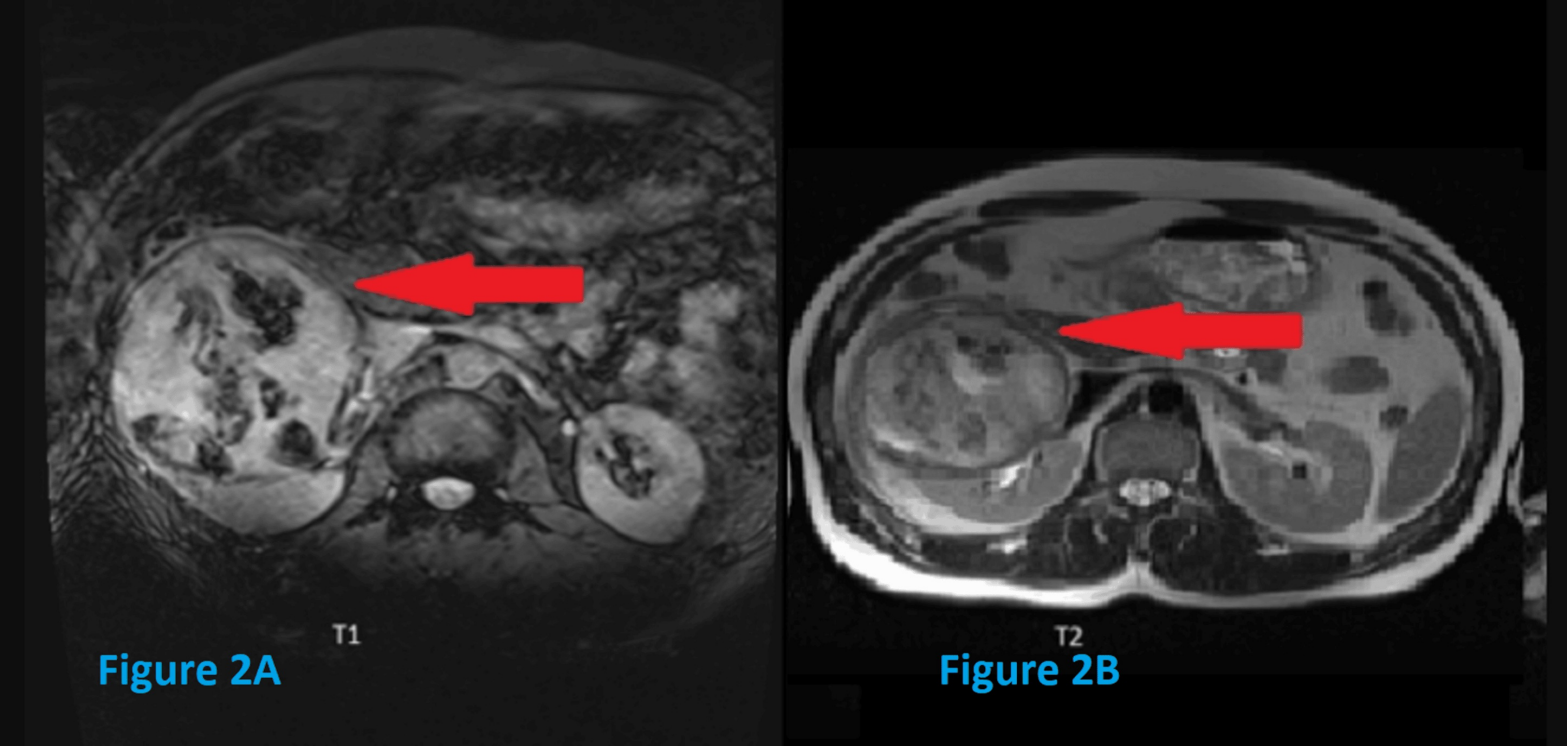
MRI images (A) A right renal lesion with multiple areas of GRE blooming, which appears hyperintense on T1 and (B) large, well-defined, heterogeneously hyperintense right renal mass on T2 weighted imaging.

As depicted in Figure 2, MRI revealed a large well-defined T2 heterogeneously hyperintense and T1 heterogeneously isointense mass lesion measuring ~ 10x9x9 cms (CC x AP x TR) arising from the mid pole of the right kidney, which showed few subtle areas of restricted diffusion and multiple areas of GRE blooming which appeared hyperintense on T1, suggestive of hemorrhagic areas. While these imaging findings are not pathognomonic for XGPN, hemorrhagic areas could be due to granulation tissue seen in XGPN [3].

Since the imaging findings were inconclusive of a tumor and in view of a high degree of suspicion, a CT-guided fine needle aspiration biopsy (FNAB) of the renal cyst was performed, which revealed a sparsely cellular haemorrhagic smear with no atypical cells. 

**Figure 3:**
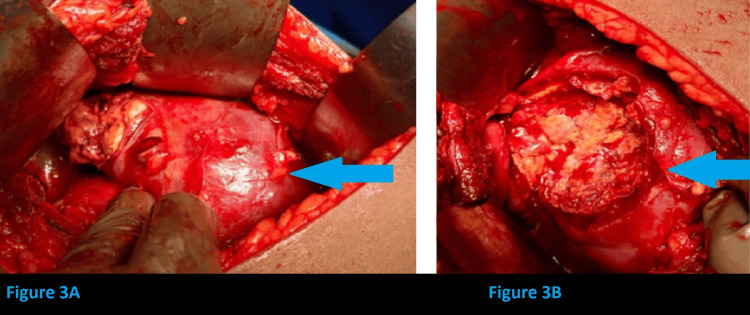
Intraoperative findings (A) Right renal cyst identified. (B) Thick, purulent material was noted inside the cyst.

**Figure 4:**
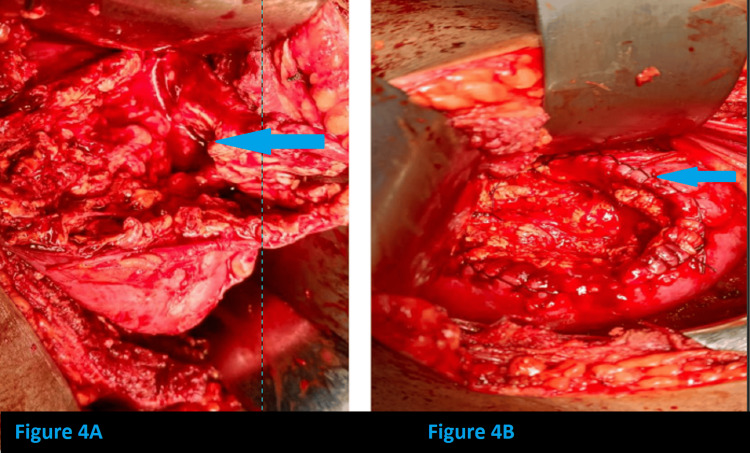
Intraoperative images (A) The sloughy and thick, tenacious purulent material of the cyst was cleared. (B) The cyst was completely excised, and closure of the renal parenchymal edges was done.

The patient was then taken up for surgery-open decortication and excision of the infected complex right renal cyst with right DJ stenting (retrograde DJ stenting using fluoroscopy). Dense adhesions were found to be present between kidney and pararenal tissue and fat. A renal cyst was identified arising from the posterior aspect of the mid-region of the right kidney, approximately 9 x 10 cm, abutting the second part of the duodenum and lower surface of the liver, slough present over the cyst. There was no communication between the renal cyst and the pelvicalyceal system. Adhesiolysis and decortication of cyst done. The sloughy and thick, tenacious purulent material of the cyst was cleared. The cyst was completely excised. Saline wash was given, and renal parenchymal edge closure was done in a continuous circular fashion. Intraoperative steps are shown in Figures 3, 4. 

The postoperative course was uneventful. The patient was discharged on the seventh postoperative day. He was followed up with a CT scan after two months, which showed no abnormality.

**Figure 5:**
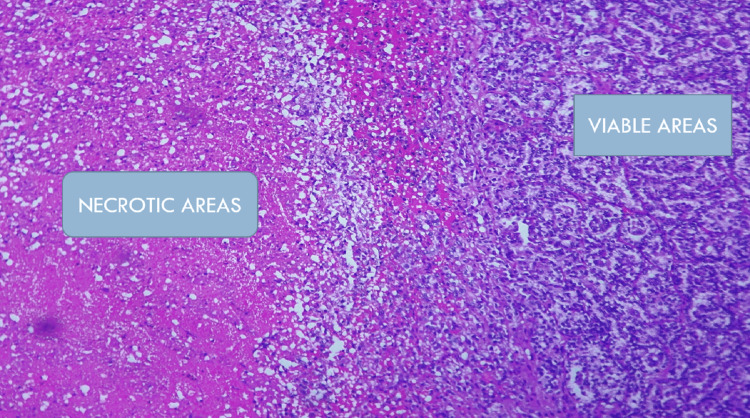
Histopathological examination of xanthogranulomatous pyelonephritis Hematoxylin and Eosin (H&E) staining at 100X magnification shows necrotic areas (on the left) and residual viable areas (on the right).

**Figure 6:**
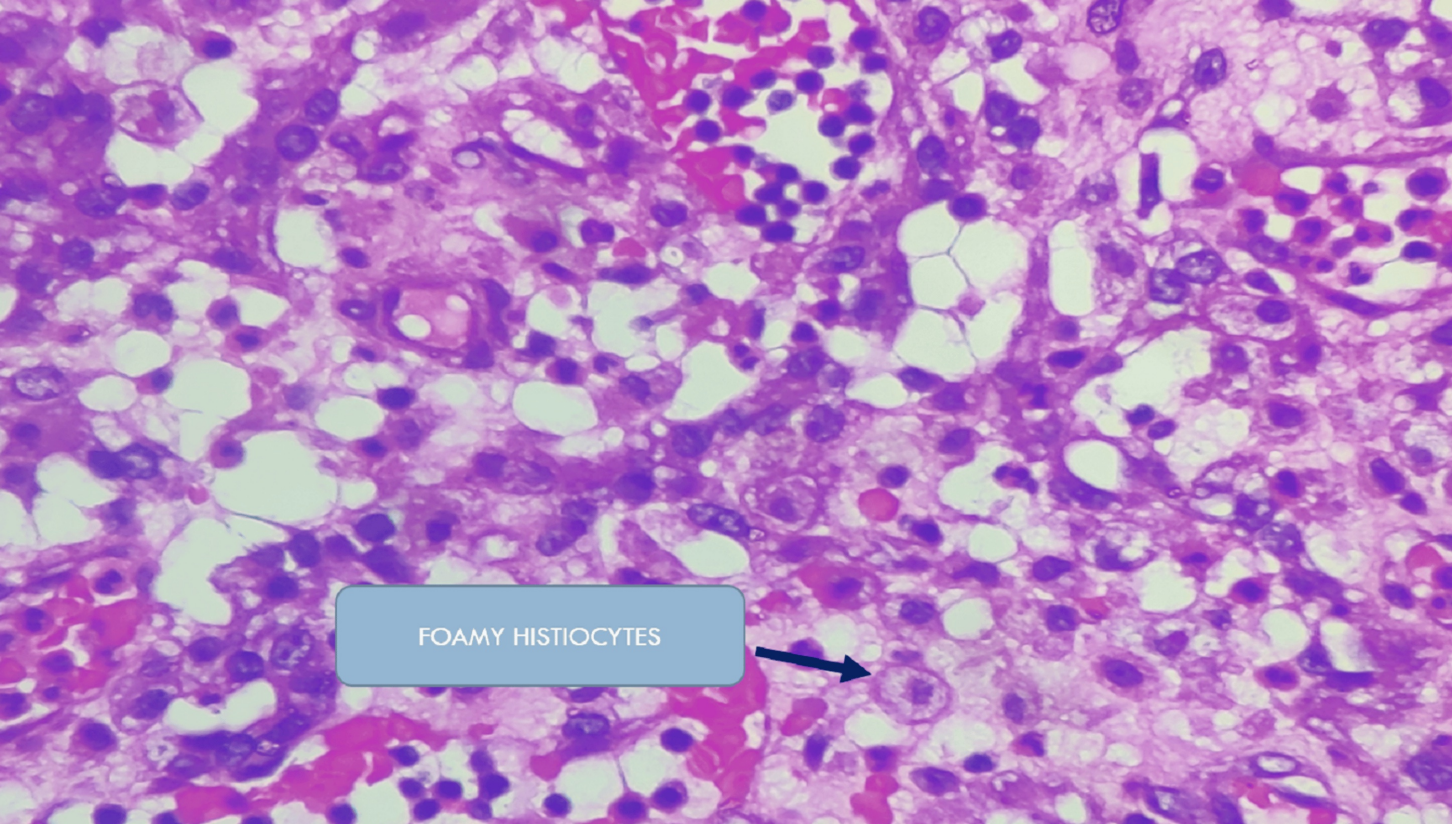
Higher magnification of xanthogranulomatous pyelonephritis Hematoxylin and Eosin (H&E) staining at 400X magnification shows an infiltrate of foamy histiocytes (arrow) interspersed between the renal parenchyma.

**Figure 7:**
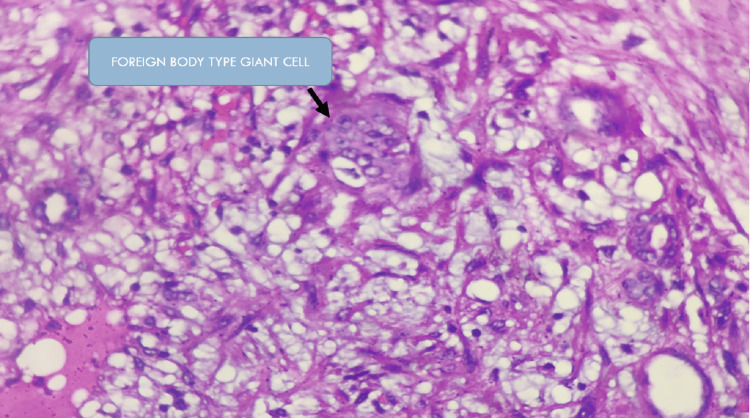
Higher magnification of xanthogranulomatous pyelonephritis Hematoxylin and Eosin (H&E) staining at 400X magnification shows occasional multinucleated foreign body giant cells (arrow) interspersed between the renal parenchyma.

Histopathological examination by Hematoxylin and Eosin stain (H&E) revealed xanthogranulomatous pyelonephritis with extensive ischaemic necrosis. The pathognomonic microscopic feature is the lipid-laden foamy macrophage accompanied by both chronic- and acute-phase inflammatory cells as shown in the Figures 5-7. Real-time tuberculosis-polymerase chain reaction (TB-PCR) test was negative. Tissue culture showed no growth.

## Discussion

Xanthogranulomatous pyelonephritis (XGPN) is a chronic granulomatous inflammatory process affecting kidneys [1]. It can present as a destructive mass, mimicking a renal malignancy in imaging studies. Although it usually presents as a diffuse or global form, it may rarely also present as a focal form [4]. XGPN is a rare but serious infection of the kidney, with an incidence of about 1.4 cases per 100,000. The diffuse form of XGPN accounts for approximately 90-92% of all XGPN cases, while the focal form of XGPN constitutes 8-10% of XGP cases [5,6]. 

XGPN commonly presents with flank or loin pain, fever, anorexia, nausea, and vomiting [7]. Positive urine cultures may be found in more than 52% of cases. The most commonly isolated bacteria are Escherichia Coli and Proteus Mirabilis [6]. Preoperative imaging identifies XGPN correctly in only about half the cases [8,9]. The diagnosis of XGPN is usually made pathologically, as there is no pathognomonic clinical or radiological sign of the disease. Treatment of the disease involves both medical and surgical intervention [10].

XGPN can be challenging to diagnose due to its variable clinical presentation and ability to mimic other renal pathologies on imaging. This case highlights the importance of considering XGPN in the differential diagnosis of a complex renal cyst, especially when clinical suspicion is high. When there is a high suspicion of a renal malignancy, the intraoperative frozen section may be considered based on the facilities available at the center [11]. ESR was mildly elevated in our case. Although an elevated ESR may be indicative of XGPN, elevated ESR in isolation is neither sensitive nor specific for the disease as it may be elevated due to any inflammatory condition [12]. C-reactive protein has a few advantages over ESR as a measure of acute phase reactants. Where possible, procalcitonin should be preferred to both CRP and ESR [13]. In our case, urine culture was positive for Enterococcus. Most commonly isolated cultures show E. coli or P. Mirabilis, or it may even be negative [14]. Appropriate antibiotics should be used for all patients with XGPN. Enterococcus is rarely implicated in XGPN [15]. FNAB of the renal cyst revealed a sparse cellular hemorrhagic smear with no atypical cells seen. This may be seen in patients with a well-differentiated renal cell carcinoma (RCC), a benign cyst, or a renal infarct. On the basis of aspirated cytology alone, it is not possible to differentiate such cases [16]. It should noted that FNAB may be non-diagnostic in as many as 30% of the samples, and a repeated FNAB may be helpful in about 60% of these cases [16]. 

Risk factors for XGPN include diabetes mellitus, immunocompromised status, obstructive uropathy, nephrolithiasis, and chronic inflammatory conditions [17,18]. Our case was well nourished, moderately built, non-diabetic, non-immunocompromised, and he did not suffer from urolithiasis. Thus, the preoperative suspicion of XGPN in this particular patient was initially obscured. Although our patient did not have any of the classical risk factors for XGPN, it is possible that he might have had a renal cyst, which subsequently got infected and later developed into XGPN [19].

Surgical approaches for the management of XGPN include open nephrectomy, open partial nephrectomy and laparoscopic nephrectomy. Open nephrectomy has long been considered the standard treatment for XGPN, which aims to remove all involved granulomatous tissue. Partial nephrectomy may be considered if the disease is limited, especially in children, to preserve residual renal function [20]. Laparoscopic nephrectomy can be a reasonable option for experienced teams. It can be less painful, cost-effective, and have better cosmetic results than open surgery [10]. This patient was managed with nephron-sparing surgery with no adverse event, thus demonstrating the efficacy of nephron-sparing surgery in patients with focal XGPN.

## Conclusions

This case report demonstrates a case of XGPN initially presenting as a complex renal cyst without typical risk factors for XGPN. This case also demonstrates the feasibility of nephron-sparing surgery, thus preventing the need for nephrectomy. Thus early recognition and appropriate management of XGPN are crucial to prevent this potentially fatal condition and preserve renal function.
